# Minocycline inhibition of microglial rescues nigrostriatal dopaminergic neurodegeneration caused by mutant alpha-synuclein overexpression

**DOI:** 10.18632/aging.103440

**Published:** 2020-07-24

**Authors:** Yong Wang, Qian Wang, Ruobing Yu, Qi Zhang, Zhonghai Zhang, Haiying Li, Chao Ren, Rongli Yang, Haichen Niu

**Affiliations:** 1 Department of Neurology, First Affiliated Hospital of Soochow University, Suzhou 215006, China; 2 Affiliated First Clinical College of Xuzhou Medical University, Xuzhou 221004, China; 3 Department of Geriatric Medicine, Affiliated Hospital of Xuzhou Medical University, Xuzhou 221004, China; 4 Department of Genetics, Xuzhou Medical University, Xuzhou 221004, China; 5 Experimental Animal Center, Xuzhou Medical University, Xuzhou 221004, China; 6 Deprtment of Physiology, Xuzhou Medical University, Xuzhou 221004, China; 7 Department of Pathology, Xuzhou Medical University, Xuzhou 221004, China; 8 Department of Neurology, Affiliated Yantai Yuhuangding Hospital of Qingdao University, Yantai 264000, China; 9 Public Experimental Research Center of Xuzhou Medical University, Xuzhou 221004, China

**Keywords:** Parkinson’s disease, α-synuclein, lewy-like pathology, IL-1β, microglia, neuroinflammation

## Abstract

Studies indicate that mutant α-synuclein (mαSyn) is involved in the pathogenesis of Parkinson’s disease (PD). The mαSyn expression leads to the loss of dopaminergic neurons in the substantia nigra (SN) and consequent motor dysfunctions. Additionally, studies found that PD was accompanied by extensive neuroinflammation of SN. However, it remains unclear as to whether microglia participate in the mαSyn pathology. This issue is addressed by using AAV-mα-Syn (A30P-A53T) to overexpress the human mαSyn in the SN in view of establishing the PD model. Subsequently, minocycline (Mino) was used to inhibit microglia activity, and an interleukin-1 receptor (IL-1R1) antagonist was used to hinder the IL-1R1 function. Finally, immunohistochemistry was used to analyze phosphorylated αSyn (Ser129) and TH-positive cells in the SN. Dopamine levels were analyzed by high performance liquid chromatography. mαSyn overexpression in the SN induced motor dysfunction, decreased striatal dopamine levels, and increased pathological αSyn 12 weeks after AAV injection. The data demonstrated that inhibiting microglial activation or hindering IL-1R1 reversed the persistent motor deficits, neurodegeneration of the nigrostriatal dopaminergic system, and development of Lewy body pathology caused by human mαSyn overexpression in the SN. Additionally, these findings indicate that neuroinflammation promotes the loss of neuronal cells.

## INTRODUCTION

Parkinson’s disease (PD) is a progressive neurodegenerative disorder characterized by motor symptoms such as bradykinesia, resting tremor, and cogwheel rigidity. Pathological studies reported the loss of dopaminergic (DA) neurons in the substantia nigra (SN) and the formation of Lewy bodies and Lewy neurites upon the autopsy of PD patients [1]. However, the etiology of this disease remains unknown.

Studies indicate that several risk factors, including harmful environmental factors and deficient genetic factors, play a vital role in sporadic PD [1]. Environmental factors, such as exposure to MPTP, lipopolysaccharide (LPS), rotenone, or other organic chemicals, can directly cause inflammation in neural tissues, leading to the loss of dopaminergic neurons [2–5]. For example, administering LPS to the striatum develops PD-like motor symptoms in mice [6]. However, dopaminergic neuron loss in the SN is accompanied by microglial activation and the release of copious amounts of nitric oxide (NO), tumor necrosis factor-α (TNF-α), interleukin-1β (IL-β), and other proinflammatory cytokines [7].

Previous studies indicated that duplications, triplications, or missense mutations (such as A53T or A30P) of the alpha-synuclein (αSyn) gene (*SNCA*) cause familial forms of PD [8, 9]. These findings indicate that mutant α-synuclein (mαSyn) is a vital pathogenic molecular marker. Misfolded αSyn leads to intracytoplasmic eosinophilic inclusions—the so-called Lewy bodies—that are a characteristic of neuropathological hallmark in PD and are spatiotemporally distributed in specific brain regions [10]. Studies have reported that mαSyn is also linked to microglial activation [11] *in vitro* to increase proinflammatory molecules [12]. Furthermore, both wild-type and mutant αSyn (A53T) can activate primary cultured microglia [13]. Meanwhile, αSyn monomers and fibrils induce the interleukin 1β (IL-1β) release *in vivo* from monocytes and microglia [14]. However, it is unknown whether the inflammation results from the mαSyn pathology. In other words, the question remains whether perpetuating microglial activation promotes PD.

To shed light on the relationship between αSyn pathology and neuroinflammation, we used a validated PD transgenic mouse model that overexpresses human mαSyn (A53T, A30P, heretofore hmαSyn) in the SN via recombinant adeno-associated virus (rAAV). Our data showed that hmαSyn overexpression in the SN caused nigrostriatal dopaminergic neurodegeneration and abnormal motor behaviors. Notably, Mino-mediated microglial inhibition enhanced nigrostriatal dopaminergic neurodegeneration. In addition, our findings revealed that inhibiting neuroinflammation can prevent hmαSyn pathology and suggested that IL-1β/IL-1R1 could be therapeutically exploited as a novel target for PD treatment.

## RESULTS

### Overexpression of hmαSyn promoted microglial activation

**Figure 1:**
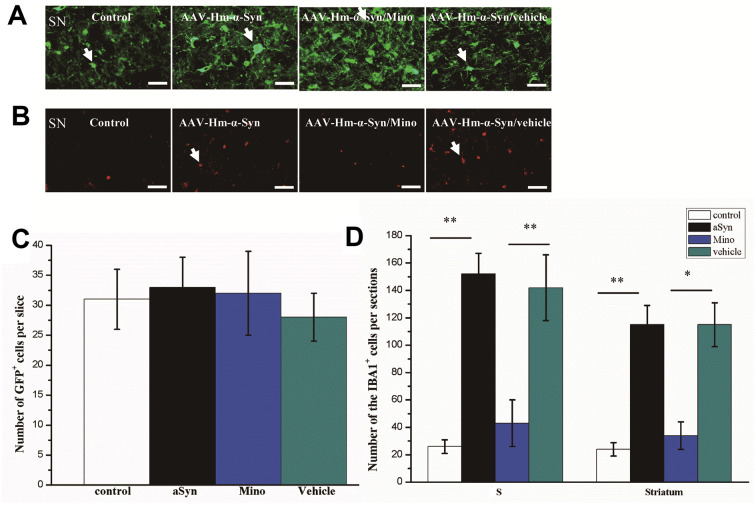
**Mino inhibited microglial activation in the striatum and substantia nigra (SN) of mice injected with AAV hm-αSyn.** (**A**) Mutant-αSyn-GFP expression after AAV SN injection in different groups. (**B**) Iba1 expression in different groups. (**C**) Quantification of mutant-αSyn-GFP expression after Mino treatment; there was no difference among the groups. (**D**) Mino inhibited Iba1 expression in the SN and striatum, as denoted by the number of Iba1^+^ cells in the SN. Data are expressed as the mean ± standard deviation (SD); **P < 0.01; *P < 0.05.

Our first objective was to determine whether hmαSyn overexpression via AAV injection in the SN would promote microglial activation. Twelve weeks after the injection, the AAV-hm-αSyn-GFP-injected mice expressed hmαSyn-GFP in SN neurons, whereas the mice that received the control injection expressed GFP (Figure 1A, 1C). A difference was noted in the number of fusion proteins in the SN among the different groups (*P* > 0.05). Furthermore, the number of Iba1^+^ cells was increased in the AAV-hm-αSyn compared to the control mice. These data indicated that the microglial cells were activated by hmαSyn and not the control injection (Figure 1B, 1D). Nevertheless, no significant difference was found between control and Mino treatment, although that seemed the more Iba1+ cells than control group.

Moreover, the elevated number of Iba1^+^ cells was reduced by Mino administration after the AAV-hm-αSyn treatment (Mino/mutant-α-Syn versus mutant-αSyn, *P* < 0.05).

### Mino administration prevented the dopaminergic neurons loss in the nigrostriatal system after hmαSyn overexpression

**Figure 2:**
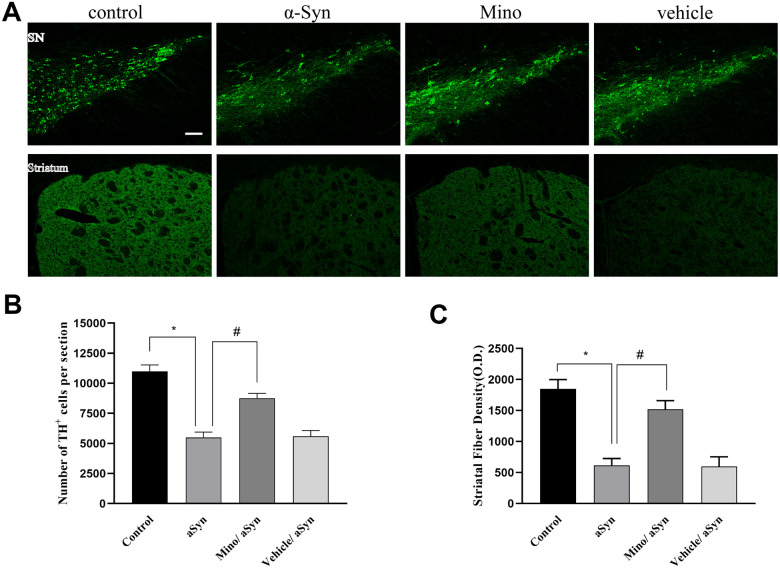
**Mino administration after AAV injection prevented the loss of dopaminergic cell bodies in the substantia nigra (SN) and striatal fibers.** (**A**) Immunohistochemical staining of the tyrosine hydroxylase (TH)^+^ cells in the striatum and SN. (**B**, **C**) Quantification of TH^+^ cells in SN and fibers in striatum. Data are expressed as the mean ± standard deviation (SD). *P < 0.05; **P < 0.01.

Our data indicated that there was widespread expression of hmαSyn in the SN 12 weeks after AAV-hm-αSyn injection. Notably, hmαSyn related to GFP as a marker. To explore whether Mino exerted a protective role on the dopaminergic neurons, we quantified the TH^+^ cells bodies in the SN and striatal fibers after Mino treatment (Figure 2A, 2B). The number of TH^+^ neurons in the SN was significantly lower in AAV-hm-αSyn-treated than in control mice (5503 ± 451 cells and 11045 ± 534 cells, respectively; *P* < 0.01). However, the number of TH^+^ cells in SN was enhanced by Mino treatment (8750± 412; *P <* 0.05). Additionally, we investigated the relative optical density of TH^+^ striatal dopaminergic fibers; in AAV-hm-αSyn-injected mice, this value was 30% relative to the control animals (*P* < 0.01). However, the striatal TH^+^ optical density after Mino treatment was increased 2.5-fold (*P* < 0.05) relative to the AAV1/2 mutant alpha-Syn group. Thus, Mino administration prevented the dopaminergic neuron loss that was promoted by hmαSyn overexpression.

### Mino administration increased the dopamine level in the nigrostriatal system after hmαSyn overexpression

**Figure 3:**
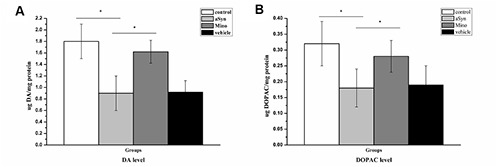
**Mino enhanced the dopamine and 3,4-dihydroxyphenylacetic acid (DOPAC) levels in the nigrostriatal system after hmαSyn overexpression.** Mino administration reversed the significant DA (**A**) and DOPAC (**B**) reduction caused by hmαSyn overexpression. Bars represent the mean ± standard deviation (SD). *P < 0.05, **P < 0.01.

HPLC was employed to evaluate the striatal dopamine and DOPAC levels. The results revealed a significant reduction of striatal DA level to 50% relative to the control group (Figure 3A; *P* < 0.01) 12 weeks after the AAV-mutant-αSyn injection. Moreover, DOPAC was significantly reduced to 44% of the control group (Figure 3B; *P* < 0.01) 12 weeks after the AAV injection in the SN. Mino treatment enhanced the DA (p < 0.05) and DOPAC (p<0.05) levels (Figure 3). Thus, Mino treatment during the mutant-αSyn overexpression in the SN inhibited the reduction of the dopamine neurotransmitter.

### Mino administration reversed the behavioral deficits induced by hmαSyn overexpression in the substantia nigra

**Figure 4:**
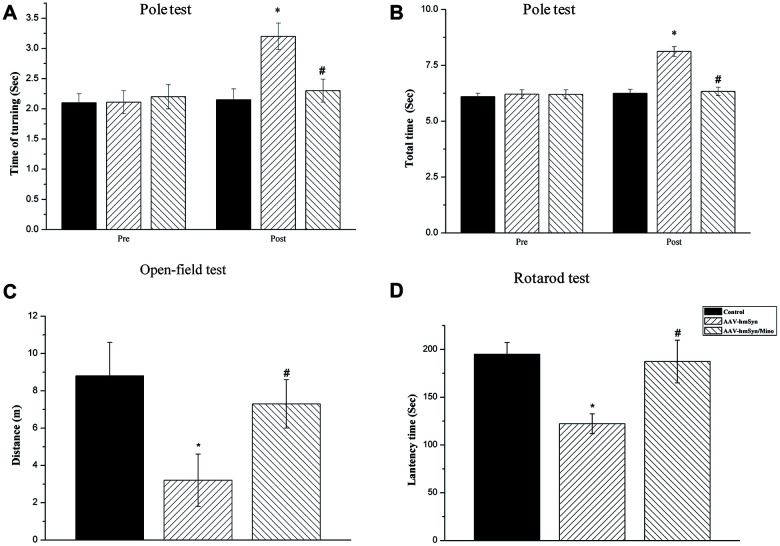
**Mino administration could inhibit the motor and non-motor deficits.** (**A**, **B**) Mino administration effects on the behavior in the pole test. (**C**) Mino administration effects on the behavior in the open-field test. (**D**) Mino administration effects on the behavior in the rotarod test. *P < 0.05 vs. control. # *P* < 0.05 vs. AAV-hm-αSyn.

To examine whether hmαSyn overexpression in the SN altered behavior in the mice, we assessed motor behavior by the pole test, rotarod test, and open field 12 weeks after AAV injection. hmαSyn overexpression resulted in a deficit in the pole test including a reduced total time and turning time. However, Mino injection reversed this deficit, leading to a 15% improvement in the time to turn (Figure 4A) and a 20% improvement in the time to descend (Figure 4B). Furthermore, the open-field was used to test the motor ability of mice by different treatments. Twelve weeks after the AAV injection, the AAV-hm-αSyn injection into the SN decreased the total distance travelled compared to control mice. As shown in Figure 4D, Mino treatment reversed the motor function deficit caused by AAV-hm-αSyn injection into the SN. These data suggest that the motor ability of mice was impaired by hmαSyn overexpression in the SN, but these deficits were reversed by Mino treatment. Finally, to assess the effect of activated microglia on motor coordination and balance, we tested the AAV-hm-αSyn, AAV-GFP, or AAV-hm-αSyn/Mino mice on an accelerating rotarod 12 weeks after the AAV injection. The data were analyzed to compare the latency to fall at different rotation speeds (Figure 4C). AAV-hm-αSyn mice spent less time on the rotarod apparatus than control mice (AAV-GFP). Mino injection significantly delayed the latency to fall on the rotarod apparatus (*P* < 0.01), and these mice performed similarly to control (AAV-GFP) mice.

### Mino inhibited IL-1β release from microglia that was activated by hmαSyn overexpression

**Figure 5:**
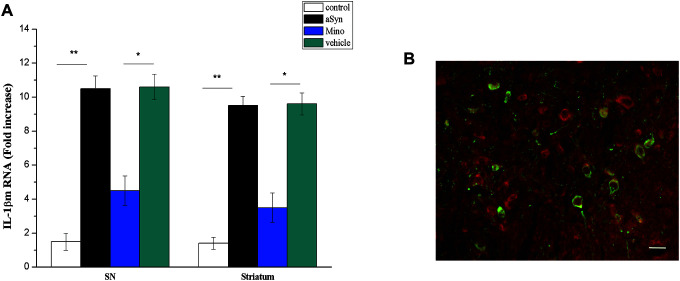
**Mino administration inhibited IL-1β release.** (**A**) Qualitative analysis of IL-1β in the SN. *P < 0.05; **P < 0.01. (**B**) IL-1 receptor immunostaining in dopaminergic neurons in the substantia nigra (SN).

Studies suggested that sustained activated microglia release various cytokines, including IL-1β, that impair neuronal functions [15]. LPS-activated microglia cause dopaminergic neuron attenuation in an IL-1-dependent manner—a phenomenon that results in PD-like behavioral impairment. Thus, we quantitated the IL-1β mRNA level in the nigrostriatal system. Changes in pro-inflammatory mRNA levels of the striatum and SN were examined in mice 12 weeks after AAV SN injection. hmαSyn overexpression enhanced IL-1β mRNA levels in the striatum (P < 0.05) and SN (P < 0.05) compared to the control mice (Figure 5A). Notably, Mino injection prevented the enhanced IL-1β mRNA expression induced by hmαSyn overexpression (P < 0.01).

We also examined IL-1β receptor expression in the SN dopaminergic neurons with immunostaining. We noted colocalization between RFP and TH staining in SN neurons. According to quantitative analysis, the number of IL-1R1^+^/TH^+^ cells was significantly increased (P > 0.05), thus indicating hm-αSyn overexpression in the SN induced IL-1R1 expression (Figure 5B). This result further suggests that the dopaminergic neuronsdopaminergic neurons of the SN field could become a target of IL-1β and might be impaired by chronic IL-1β release.

### Mino administration decreased the Lewy body pathology in the SN

**Figure 6:**
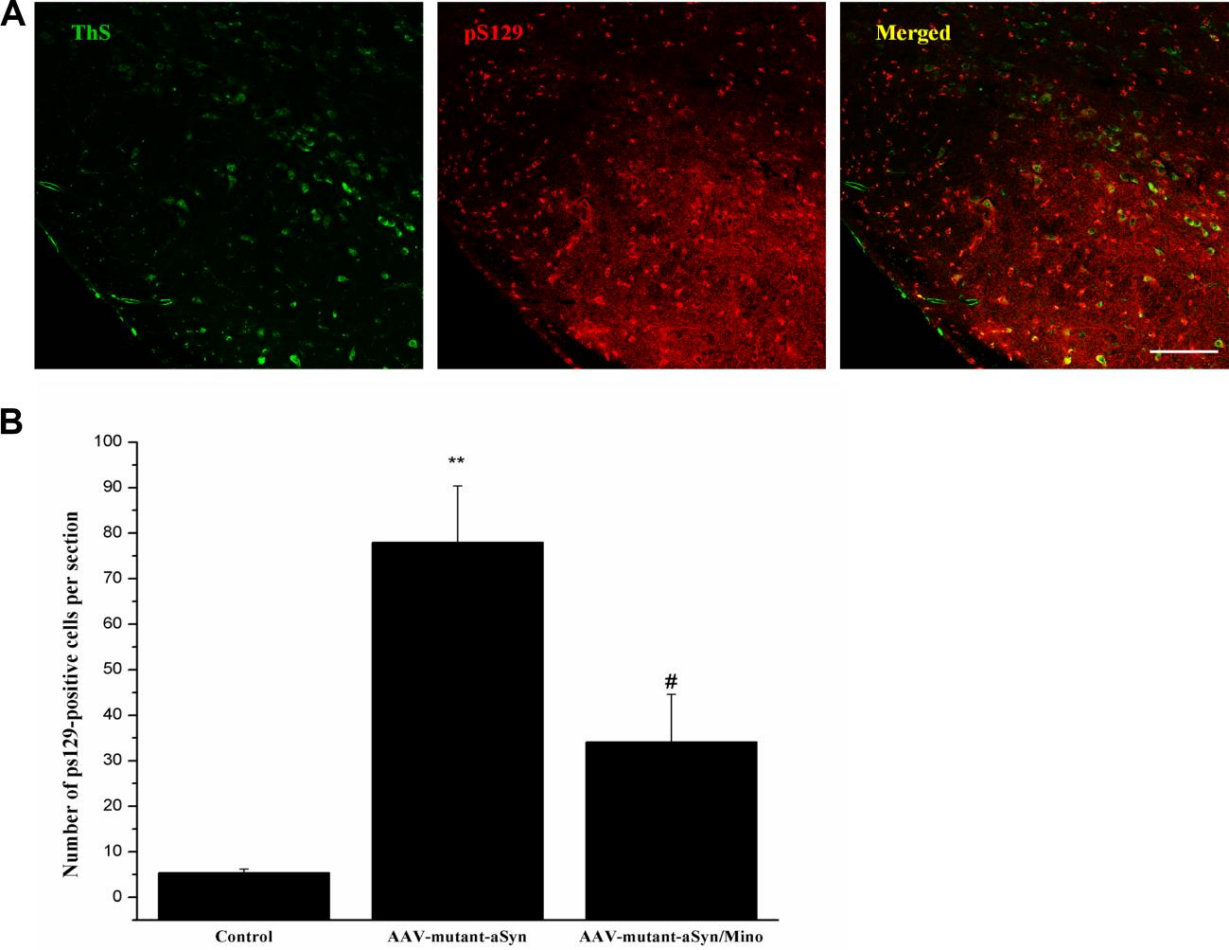
**Mino administration decreased the Lewy-body pathology in the substantia nigra (SN).** (**A**) Mino decreased the pS129 αSyn signal induced by hmαSyn overexpression in the SN. (**B**) Quantitative analysis for the pS129 signals in the SN.

To observe the toxic effects of hmαSyn, the pS129 αSyn was detected in the SN using immunostaining (Figure 6). hmαSyn overexpression enhanced pS129 αSyn compared to the control mice (*P* < 0.05). Mino treatment decreased the enhanced pS129 signal in the SN compared to AAV-mutant-αSyn treatment (*P* < 0.05). Furthermore, the pS129 signal was confirmed by the ThS staining that was used to explore Lewy bodies. In general, the data indicate that the AAV-mutant-αSyn injection might induce the αSyn pathology in the SN and that the inhibition of microglia by Mino treatment prevents the αSyn pathology caused by hmαSyn overexpression.

## DISCUSSION

This study aimed to explore whether the neuroinflammation contributed to αSyn pathology. In the experiments, the AAV vector that contained human mutant A30P-A53T-αSyn was bilaterally injected into the SN to induce mutant αSyn overexpression. This treatment led to the loss of dopaminergic cell bodies and fiber terminals, followed by a decrease in DA in the striatum, and abnormal motor functions (common to PD) 12 weeks after AAV injection. Histopathological studies also demonstrated that the number of Lewy bodies increased in certain brain regions. However, we inhibited microglial function by administrating Mino, an action that reversed the effects of hmαSyn overexpression. Furthermore, IL-1β released from activated microglia played a vital and necessary role in the pathology mediated by αSyn. Mino treatment inhibited microglial function and prevented the pathology induced by overexpression of hmαSyn in the SN. In general, all data indicated that microglia modulated the pathology via IL-1β, which stimulates its receptor in neural cells.

Moreover, previous studies reported that hmαSyn overexpression can be caused by AAV in mice and rats [15–18]. hmαSyn, under the cytomegalovirus (CMV) promoter, was overexpressed in the SN. In these neurons, hmαSyn could interact with endogenous αSyn and transform the normal form into pathological αSyn [19]. We employed ThS and pS129 signals to detect pathological αSyn or Lewy bodies/neurites in the SN, which were the “gold standard” histological methods. Results indicated that the colocalization between ThS and pS129 signals in SN was found, followed by the hmαSyn overexpression. This result was also in line with the Lewy bodies observed in humans. Evidence indicates that misfolded αSyn can be transformed into Lewy bodies that are labelled by the pS129 αSyn antibody [16]. HmαSyn overexpression in the SN destroyed dopaminergic cell bodies and terminals, denoted by the reduced number of TH^+^ cells in the SN and the decreased striatal fiber density. Besides, dopamine and DOPAC levels were significantly reduced [16, 20]. The motor and non-motor deficits were found 12 weeks after AAV injection. Previous studies implied that hmαSyn expression in SN induces motor impairment in different PD animal models [16, 21].

AAV injection induced hmαSyn overexpression in neuronal and non-neuronal cells, including microglia. Twelve weeks after injection, the number of GFP-positive cells in the SN was not significantly different between AAV-control and AAV-hm-αSyn groups. However, there were more activated microglia marked by Iba1/CD11b in the AAV-hm-αSyn than in the control group. These results indicated that AAV-hm-αSyn injection activated more microglia. Additionally, inhibiting microglia with Mino treatment reduced the loss of dopaminergic neurons; this indicates that deactivating microglia might inhibit the pathology exhibited by neuronal cells [22].

The activated microglia released some pro-inflammatory cytokines, including IL-1β [23]. Under physiological condition, IL-1R1 is not found in the SN (unpublished data); however, hmαSyn overexpression also led to IL-R1 expression in SN. Notably, the IL-1β–IL-R1 system caused the αSyn pathology. Specifically, hmαSyn overexpression elevated the IL-1β mRNA level in SN compared to control treatment; such finding is consistent with a previous study [24]. Another study indicated that misfolded hmαSyn could be transformed into fibril αSyn that could be transmitted among cells and lead to microglial activation and consequent IL-1β release [19]. hmαSyn overexpression also produced IL-1R1 expression in SN dopaminergic neurons. This change was coupled with IL-1β from activated microglia. Therefore, IL-1β from activated microglia and its receptor in the dopaminergic neurons played a vital role in the pathology of hmαSyn.

## CONCLUSIONS

Our data demonstrated that the mice injected with AAV-hm-αSyn in the SN met several criteria required for PD model. Furthermore, activated microglia seem to play a vital role in PD pathogenesis. However, Mino inhibited microglial function to reduce the IL-1β. This effect prevented the pathology caused by the AAV-hm-αSyn overexpression. Inhibiting inflammatory response from activated microglia might become an alternate employed to slow PD progression. To our knowledge, the IL-1β–IL-1R1 system would represent a novel target for PD treatment.

## MATERIALS AND METHODS

### Animals

Eight-week-old male C57BL/6 mice (Xuzhou Medical University, China) were used in the experiment. Breeders for the IL-1R1 reporter (IL-1R1^GR/GR^) mice were obtained from Dr. Ning Quan from Florida Atlantic University (Jupiter, USA). Age- and sex-matched littermate controls were used for experiments. IL-1R1^GR/GR^ mice were used to detect if the IL-1R1 was localized in the dopaminergic neurons in the SN. IL-1R1^GR/GR^ mice have normal IL-1R1 expression in all cell types under their endogenous promoter. The GR allele contains 3HA and IRES-tdTomato sequences at the 3′ end of the IL-1R1 gene. These sequences allow tracking IL-1R1 messenger RNA (mRNA) by tdTomato fluorescence and protein expression via the HA tag [25]. Four mice were housed per cage with *ad libitum* access to food and water under a 12 h/12 h light-dark photoperiod. All operations and animal handling in the studies followed the ethical standards of Xuzhou Medical University animal studies (protocol number: 201704221).

### rAAV vectors and stereotactic injection

A combination of rAAV serotype 1 and 2 vectors was used to express mutant-A30P-A53T-αSYN (rAAV1/2-hSyn-human-a30P-A53T-αSYN-EGFP [rAAV1/2-hmαSYN-eGFP]) or the control (rAAV1/2-eGFP) in specific neuronal cells. The hmαSyn complementary DNA (cDNA) was a gift from Dr. Shujiang Shang (Peking University) and was inserted between the promoter and an internal ribosome entry site (IRES) element of pTR-UF12 to build the construct. Real-time quantitative polymerase chain reaction (qPCR) was used to determine the number of genome copies (4 × 10^13^ genome copies/μl). All AAV packaging and purification were completed with Vigen Biosciences (Qingdao, China) products. The mice were deeply anaesthetized with 10% of chloral hydrate (0.04 ml/10 g body weight) via intraperitoneal injection. Then, either 0.5 μl rAAV1/2-eGFP (control) or 0.5 μl rAAV1/2-hmαSYN-eGFP, both at a concentration of 4 x 1012 genomic particles (gp)/ml, was bilaterally injected in the mice’s SN using a microinjector at a rate of 0.05 μl/min. Based on the mouse brain atlas of Paxinos and Franklin (Paxinos and Franklin, The Mouse Brain in Stereotaxic Coordinates, Second Edition, 2001), the SN coordinates from Bregma (AP -3.1 mm; ML -1.4 mm; DV -4.4 mm) were applied [26]. After the administration, the needle was left for 10 min before withdrawal to prevent leakage. One month after the AAV treatment, the mice were euthanized.

### Inhibiting microglial activation

Microglia were blocked by Mino administration in order to determine whether activated microglia promote the pathological process induced by hmαSyn overexpression. Mino is a clinically available tetracycline antibiotic that can cross the retinal blood barrier and exert anti-inflammatory, anti-apoptotic, and neuroprotective effects by inhibiting microglial function [27]. Minocycline hydrochloride (Sigma-Aldrich, CAS 13614-98-7) was dissolved in sterile water, and 30 mg/kg was injected once daily after AAV administration. Mino was administered after AAV injection to avoid the central inflammatory response to the hmαSyn challenge.

### Catecholamine quantification by high-performance liquid chromatography (HPLC)

HPLC was used to detect changes in the levels of dopamine and its metabolite 3,4-dihydroxyphenylacetic acid (DOPAC) in the SN 12 weeks after the AAV injection. The animals were deeply anesthetized with a combination of 120 mg/kg ketamine and 16 mg/kg xylazine. Subsequently, striatal samples were dissected and frozen in liquid nitrogen. Brain sections were homogenized in 500 μl of 0.1 M trichloroacetic acid (TCA) (1 x 10^-2^ M sodium acetate, 1 x 10^-4^ M ethylenediaminetetraacetic acid [EDTA], and 10.5% methanol) as previously described [28]. After centrifugation at 10,000 *g* for 30 min, the supernatant was removed. HPLC, coupled with electrochemical detection with an Antec Decade II, was used to analyze the catecholamine levels. Supernatant samples were injected by utilizing a Water 717+ autosampler onto a Phenomenex Nucleosil (5u, 100A) C18 HPLC column (150 x 4.60 mm). A mobile phase (75.2 mM sodium phosphate, 1.39 mM 1-octanesulfonic acid, 0.125 mM ethylene diamine tetraacetic acid, 0.0025% triethylamine, and 10% acetonitrile, pH 3.0 [adjusted with 85% phosphoric acid]) was followed by delivery of the solvent at 0.38 ml/min with a Waters 515 HPLC pump. The levels of dopamine and DOPAC were detected. Catecholamine values are expressed as ng /mg total protein.

### qPCR

Studies indicated that activated microglia release several pro-inflammatory factors, including IL-1β, that might lead to neuronal degeneration after chronic treatment [29]. We quantified mRNA levels in the SN and striatum after microglia inhibition. SN and striatal samples were dissected as previously described. Total RNA was extracted through the TRIzol (Invitrogen, Carlsbad, CA, USA) method. SN and striatal samples were homogenized in 1 ml TRIzol with an Omni 2000 tissue homogenizer. After incubation at room temperature for 5 min, 250 μl chloroform was added, tubes were vortexed, and samples were incubated for 3 min. The samples were centrifuged (12,000 *g*) for 15 min at 4° C, and isopropyl alcohol (500 μl) was added to the aqueous phase to precipitate nucleic acid. The samples were vortexed briefly and incubated for 10 min, followed by centrifugation (12,000 *g*) for 20 min at 4 °C. The precipitate was washed in 75% ethanol (1 ml) and centrifuged (7,500 *g*) for 5 min at 4 °C. The samples were air dried after ethanol was decanted, and the pellets were resuspended in 20 μl RNase-free water. UV spectrophotometric analysis of nucleic acid was performed at 260 nm to determine the final concentration. Samples were DNase-treated (DNA-free kit, Ambion) to remove contaminating DNA. qPCR was performed with the following primers: forward, 5′-GAGCTGAAAGCTCTCCACCT-3′ and reverse, 5′-TTCCATCTTCTTCTTTGGGT-3′. Gene expression was performed in a DNA thermal cycler (LC480) using the following protocol: a denaturation step at 95 °C for 2 min, followed by 40 cycles of denaturation at 95 °C for 15 s and annealing at 60 °C for 40 s. β-actin was used as an endogenous control to normalize the mRNA level for each sample. A non-template control was used as the negative control. Standard curves were generated from serial dilutions of expected products over five orders of magnitude. PCR reactions were 20 μl and contained iQ Supermix (Bio-Rad) and 5 nM fluorescein isocyanate (FITC) dye. The reaction efficiency (E) was determined from the standard curves. The threshold cycle (Ct) values were transformed using (1 + E)Ct to determine the relative differences in mRNA expression.

### Behavioral tests

### Pole test

The pole test was used to evaluate whether Mino prevented the behavioral dysfunction (bradykinesia and motor coordination) caused by hmα-Syn overexpression in the SN. For the test, a vertical wooden pole (length of 50 cm and diameter of 1 cm) was placed in the home cage. The test comprised training and test stages performed 12 weeks after the AAV injection.

Training (day 1): to habituate the mice to the environment, they were first placed head-down on the top of the pole and trained to directly walk down to their home cage from the top of the pole (three trials). Then, the mice were trained to turn 180° (head-up) at the top of the pole and to descend the pole by performing a minimum of five trials with their head up.

Training (day 2): 24 hours after training, the mice performed five trials with their head up on the top of the pole.

Test day: the turning time and total duration of the descent were recorded as previously described. All the behavioral results were video recorded. An observer, who was blind to the animal group, recorded the time and scores. Each animal was tested within five trials.

### Rotarod test

The rotarod test was used to assess motor coordination in mice. The test included pre-training and test procedures. First, the mice were pre-trained on the rotarod (Ubinlab, Beijing, China) three times—each separated by 1 h—using the accelerating mode (4 to 40 rpm over 5 min). On the test day, mice ran on the rotarod at a constant velocity of 15, 20, 25, 20, and 35 rpm for a maximum of 300 s, and the latency to fall was recorded. Data were collected from three trials separated by a 1 h interval.

### Open field test

The open field test was used to assess spontaneous exploratory activity and locomotion in mice. The test was performed using the open field working station (AnyMaze Associates, Dublin, Ireland). The mice were briefly placed individually at the center of the open-field arena (40 x 40 x 50 cm), and a video camera system was used to record the animals’ behavior for 15 min. The total distance moved, time of ambulatory movements, and time spent resting were analyzed.

### Immunohistochemistry

Mice were euthanized with sodium pentobarbital and transcardially perfused with cold 0.1 M phosphate-buffered saline (PBS; pH 7.4) followed by 4% paraformaldehyde (PFA) in 0.1 M phosphate buffer. After the perfusion, the brains were removed from the skull and post-fixed overnight in 4% PFA in 0.1M PBS and then dehydrated in a sucrose gradient (20% and 30%) until they sank to the bottom of the solution. Subsequently, the brains were frozen at -80 °C and then sectioned (30 μm) with a vibrating microtome (Leica CM1950). Free-floating sections were placed in cryoprotectant until staining. Sections were washed in 0.1 M PBS (3 × 3 mins) and blocked with 5% normal donkey serum (1% bovine serum albumin [BSA] and 0.1% Triton-X in PBS). The sections were incubated with primary antibodies overnight at 4 °C. Next, the sections were washed in PBS (3 × 3 mins) and incubated with 3,3'-diaminobenzidine (DAB) or fluorochrome-conjugated secondary antibody. Sections were mounted on slides and cover-slipped with VECTASHIELD (Vector Laboratories).

### Antibodies

The primary antibodies used for immunostaining were: rabbit anti-mouse tyrosine hydroxylase (TH; 1:500; abcam, ab112), rabbit anti-mouse Iba-1 (1:500; abcam, ab178846), rabbit anti-mouse GFAP (1:500; Abcam, ab7260), phosphorylated rabbit anti-α-syn (pS129, 1:10000, abcam, ab51253), and rabbit anti-RFP (1:400; abcam, ab62341). The secondary antibodies used for immunostaining were donkey anti-rabbit immunoglobulin G (IgG) polyclonal antibody conjugated to Alexa Fluor 488 (1:500; abcam, ab150073) or Alexa Fluor 594 (abcam, ab150076).

ImageJ (NIH) was used to determine the mean immunostaining intensity values obtained from the striatum and cortices. Striatal TH-immunoreactive fibers were quantified in ImageJ according to previous studies. The relative optical density (OD) of the striatum was determined by subtracting the mean intensity of the cortex from the mean intensity of the ipsilateral striatum and averaging these values for two separate sections from the same animal. The relative mean intensity values were contrasted with the per cent of striatal denervation.

### Thioflavin S staining (ThS)

ThS staining was performed to confirm hmαSyn aggregation in the SN after AAV injection. The staining was prepared as previously described [16]. Free-floating brain sections were washed with 0.1 M PBS (3 x 3 mins), mounted onto slides, air-dried and then rehydrated with ultrapure water. The sections were stained with 0.1% Thioflavin-S ethanol/PBS for 10 min. After being washing with 0.1 M PBS (3 x 3 mins), the sections were incubated with rabbit anti-αSyn (pS129; 1:1000; abcam, ab51253) overnight and then with donkey anti-rabbit IgG conjugated to Alexa Fluor 594 (Thermo Fisher, A-21207). Slides were then mounted using VECTASHIELD.

### Statistical analysis

For the statistical analysis, QQ plots were used to assess the distribution of each set of values. For normally distributed data, parametric methods were utilized. In cases of different variances, Welch’s correction was used, and for non-normally distributed data, non-parametric methods were employed. Data from the behavioral studies were analyzed with one-way analysis of variance (ANOVA), followed by Fisher’s least significant difference (LSD) post hoc test. For immunohistochemistry data, one-way ANOVA tests were employed, followed by Fisher’s LSD. Analyses were performed with OriginPro 8 software (OriginLab, USA). All data are presented as mean ± standard error of the mean (SEM). *P < 0.05; **P < 0.01; ***P < 0.001.

### Ethics approval

This study was carried out by the recommendations of the Animal Care Committee (ACC) guidelines at the University of Xuzhou medical university in Xuzhou. The ACC at Xuzhou medical university in Xuzhou approved all protocols.
